# Extreme Weather Injuries and Fatalities, 2006 to 2021

**DOI:** 10.1001/jamanetworkopen.2024.29826

**Published:** 2024-08-26

**Authors:** Caroline Q. Stephens, Christopher Newton, Brandon Kappy, Caroline G. Melhado, Mary E. Fallat

**Affiliations:** 1 Division of Pediatric Surgery, UCSF Benioff Children’s Hospitals, and Department of Surgery, University of California, San Francisco; 2 Division of Emergency Medicine, Children’s National Hospital, Washington, DC; 3 Hiram C. Polk Jr Department of Surgery, Division of Pediatric Surgery, University of Louisville School of Medicine, and Norton Children’s Hospital, Louisville, Kentucky

## Abstract

**Importance:**

Extreme weather has major implications for state and national health care systems; however, statistics examining weather-related injuries and fatalities are limited.

**Objective:**

To examine the frequency and regional distribution of major disaster events (MDEs) in the US.

**Design, Setting, and Participants:**

This ecologic cross-sectional study of MDEs occurring between January 1, 2006, and December 31, 2021, evaluated US data on all injuries and fatalities included in the National Oceanic and Atmospheric Administration National Centers for Environmental Information Storm Events Database. The data analysis was performed between February 22, 2023, and April 1, 2024.

**Exposures:**

Major disaster events defined as an environmental event that caused either at least 50 injuries or at least 10 deaths.

**Main Outcomes and Measures:**

All MDEs were evaluated using descriptive statistics for event type, property damage, and rural or urban classification according to the National Centers for Health Statistics Urban-Rural Classification Scheme for Counties. The location of events according to Administration for Strategic Preparedness and Response (ASPR) region and hospital bed capacity of ASPR regions were also examined.

**Results:**

Between 2006 and 2021, 11 159 storm events caused 42 254 injuries and 9760 deaths. Major disaster events accounted for 209 weather events (1.9%) but caused 19 463 weather-associated injuries (46.1%) and 2189 weather-associated deaths (22.4%). The majority of MDEs were caused by extreme heat (86 [41.1%]) and tornadoes (67 [32.1%]). While a larger proportion of MDEs occurred in urban areas (151 [75.1%]) vs rural areas (50 [24.9%]), rural MDEs caused a median of 9 (IQR, 2-16) deaths per event vs 4 (IQR, 0-14) deaths per event in urban areas. The majority of MDEs occurred in either ASPR region 4 (51 [24.5%]) or region 9 (45 [21.6%]). Certain event types, such as fires, wind, and hurricanes or storms, were geographically concentrated, while extreme heat and floods affected regions across the US equally. Urban counties had disproportionately greater hospital bed capacity than rural counties relative to population and MDE distributions.

**Conclusions and Relevance:**

The findings of this ecologic study indicate that while MDEs accounted for a small proportion of all weather events, they were associated with a disproportionate number of injuries and fatalities. Integrating these data into county, state, and regional hazard vulnerability analyses is crucial to ensuring preparedness and mitigating climate risk.

## Key Points

**Question:**

How often are annual climate events associated with sufficient injury or fatality to overwhelm US health systems?

**Findings:**

In this ecologic cross-sectional study of 11 159 storm events between 2006 and 2021, major disaster events causing at least 50 injuries or at least 10 deaths accounted for 1.9% of total weather events but 46.1% of injuries and 22.4% of deaths among all regions of the US.

**Meaning:**

Given this outsized influence of major disaster events on the US health care system, these events must be tracked to ensure preparedness.

## Teaser

This cross-sectional study examines the frequency and regional distribution of major disaster events in the US.

## Introduction

The increased frequency of extreme climate events has been shown to be associated with mental, physical, and spiritual health.^1^ These outcomes are well described in the US National Climate Assessment, which occurs every 4 years and provides a scientific foundation to support decision making regarding the outcomes, risks, and vulnerabilities of the changing climate.^2^ Despite the increased recognition of the health impacts of climate change, most research on climate and human health has focused on the specific contributions of extreme heat and infectious diseases. Missing from this discussion are the traumatic injuries and deaths that occur during climate events. Despite the comprehensive nature of the National Climate Assessment, the report has not acknowledged the broader implications that an increase in climate event intensity and frequency has on the potential for traumatic injury and the attendant challenges that climate poses to trauma system preparedness.

Injuries and fatalities become a concern for health system functioning when the number of traumatic injuries that result from a disaster exceeds the capacity and capability of these systems to triage and transfer critically ill and injured patients efficiently. These challenges may be exacerbated by property damage that renders local health facilities inoperable. Previous work outside the US has developed definitions to quantify disaster events that cause sufficient injuries and fatalities to overwhelm health systems as those that cause at least 10 deaths or at least 50 injuries.^3^ These definitions have not been applied to US disasters, limiting national, regional, and state trauma system preparedness.

The National Centers for Environmental Information (NCEI), under the National Oceanic and Atmospheric Association (NOAA), maintain the Storm Event Database (SED), which documents “storms and other significant weather phenomena having sufficient intensity to cause loss of life, injuries, significant property damage, and/or disruption of commerce.”^4^ Our primary objective was to use SED data to determine what types of climate events cause sufficient injuries and fatalities to potentially overwhelm US health systems. Our secondary objective was to assess the rurality and regional distribution of the climate events that cause significant injury and fatality. We hope that these data can inform region-specific risks, aiding climate-related preparedness and mitigation efforts.

## Methods

This ecologic cross-sectional study of major disaster events (MDEs) was conducted between January 1, 2006, and December 31, 2021, using the SED data. All events with associated injuries or fatalities were included. Any event with no recorded injuries or fatalities was excluded. An MDE was defined as any event with at least 50 associated injuries or at least 10 associated deaths.^3^ Storm events were divided into 2 groups: MDEs and non-MDEs. The University of California, San Francisco, Institutional Review Board certified this study as exempt category 4 secondary research for which consent is not required. The Strengthening the Reporting of Observational Studies in Epidemiology (STROBE) reporting guideline was used; no specific guidelines exist for ecologic studies.

The National Weather Service is the primary data source for the SED. It receives data from federal emergency management, states, counties, National Weather Service damage surveys, law enforcement, newspapers, the insurance industry, and the public.^5^ The SED consists of 3 components: an event dataset, a fatalities dataset, and a location dataset. The primary dataset used in this study was the event dataset, which includes the date, state and county, event type, associated property damage, associated crop damage, the aggregate number of associated injuries (indirect and direct), the aggregate number of associated fatalities (indirect and direct), and event narrative from which the data were obtained. Municipalities must report fatality data for deaths to be included in the dataset. No individual-level demographic data are provided regarding these injuries or fatalities beyond the event narrative. Event types were grouped into flood, current, or tide; hurricane or storm; extreme heat; fires; tornado; cold, ice, or winter weather; wind; and other.

To determine which US regions were most affected by climate events, we grouped US geography by Administration for Strategic Preparedness and Response (ASPR) regions. We chose to use the ASPR categorizations because ASPR funds multiple nationwide disaster preparedness and mitigation programs and is housed under the US Department of Health and Human Services, linking it to health care systems. Conversely, the Federal Emergency Management Agency (FEMA) is housed under the US Department of Homeland Security. It primarily engages in disaster response and recovery, focusing on community rather than health system preparedness. To allow for assessment of rural vs urban impact, SED data were linked to the National Centers for Health Statistics Urban-Rural Classification Scheme for Counties.^6^ A binary definition of rurality was used, collapsing the 4 metropolitan groups into 1 urban cohort and the 2 nonmetropolitan groups into a rural cohort. Thus, any county with a metropolitan statistical area of at least 50 000 people was defined as urban, and any county with a metropolitan statistical area of 49 999 people or fewer was defined as rural. The data were divided into the associated ASPR region by state. National Centers for Health Statistics urban vs rural county classifications were superimposed onto census tract populations and land area densities (number of people per square kilometer).^7^ For comparison against existing health care capacity in these areas, statewide bed capacity of short-term, critical access, and children’s hospitals was taken from facility-level hospital use data provided to the Department of Health and Human Services.^8^

The definition of an MDE used in this study was derived from a prior Delphi survey conducted in Korea to create consensus around research frameworks for disasters.^3^ In this Delphi survey, any event with at least 10 deaths or at least 50 injuries was determined to have the potential to overwhelm the health system by requiring increased medical resources over what is available for everyday needs. This definition was used due to a lack of existing definitions in the US for determining when climate disaster events reach a threshold of injury that may overwhelm health systems.

### Statistical Analysis

Descriptive statistics were used to examine event type and regional distributions. We conducted the statistical analysis between February 22, 2023, and April 1, 2024, using Stata, version 17 (StataCorp LLC) software.

## Results

### MDE Characteristics

Between 2006 and 2021, 11 159 storm events caused 42 254 injuries and 9760 deaths. While only 209 storm events (1.9%) were MDEs, these events accounted for 46.1% (n = 19 463) of injuries and 22.4% (n = 2189) of deaths, or approximately 1216 injuries and 137 deaths per year. The most common MDEs were extreme heat (86 events [41.1%]) and tornadoes (67 events [32.1%]) (Table 1). There were 2 MDEs coded as other, both of which were caused by debris flow, a type of severe landslide.^9^ The median number of injuries per MDE was 60 (IQR, 0-119) and the median number of MDE fatalities was 6 (IQR, 1-14), compared with a median of 1 injury (IQR, 0-2) per event and 1 fatality (IQR, 0-1) per event for all events combined. A median of 12 (IQR, 8-14) MDEs occurred annually. The frequency of MDEs varied over time, with the largest number of events in a single year being 39 in 2011 vs the lowest being 3 in 2019. The MDEs most often occurred in the spring and summer, with April, July, and August averaging 2 or more MDEs per month.^9^

**Table 1.** Median Property Damage, Injuries, and Fatalities From MDEs vs Non-MDEs

Event	No. of events (%)		Median (IQR)					
			Property damage, $		Injuries, No.		Deaths, No.	
	Non-MDE	MDE	Non-MDE	MDE	Non-MDE	MDE	Non-MDE	MDE
All event types	10 950 (98.1)	209 (1.9)	0 (0-70 000)	50 000 (0-50 000 000)	1 (0-2)	60 (0-119)	1 (0-1)	6 (1-14)
Flood, current, or tide	2264 (20.7)	16 (7.7)	0 (0-15 000)	24 500 000 (10 000-800 000 000)	0 (0-1)	0 (0-50)	1 (1-1)	11 (5-20)
Hurricane or storm	1775 (16.2)	5 (2.4)	0 (0-0)	1 130 000 000 (500 000 000-3 880 000 000)	1 (1-2)	64 (0-121)	0 (0-1)	2 (1-13)
Extreme heat	1338 (12.2)	86 (41.1)	0 (0-0)	0 (0-0)	0 (0-1)	50 (0-130)	1 (0-1)	9.5 (1-16)
Fires	355 (3.2)	6 (2.9)	20 000 (0-500 000)	10 500 000 (0-75 000 000)	2 (1-3)	10 (0-61)	0 (0-1)	14 (8-19)
Tornado	1504 (13.7)	67 (32.1)	500 000 (100 000-2 500 000)	48 000 000 (14 100 000-160 000 000)	2 (1-5)	75 (54-121)	0 (0-0)	6 (2-14)
Cold, ice, or winter weather	1081 (9.9)	19 (9.1)	0 (0-0)	1 200 000 (0-33 700 000)	0 (0-1)	64 (53-115)	1 (1-1)	0 (0-2)
Wind	2565 (23.4)	8 (3.8)	15 000 (0-75 000)	32 500 (0-8 450 000)	1 (1-2)	60 (25-150)	0 (0-1)	1 (0-11)
Other^a^	68 (0.6)	2 (1.0)	20 000 (0-100 000)	339 000 000 (60 000 000-617 000 000)	1 (0-5)	90 (12-168)	0 (0-1)	32 (21-43)

Abbreviation: MDE, major disaster event.

^a^ Debris flow (a type of severe landslide).

The median costs of property damage and number of injuries and fatalities caused by each event type are shown in Table 1. All MDE weather event types except for heat and wind caused a median of at least $1 million in property damage. The single event with the greatest injury and fatality was a tornado in Missouri on May 22, 2011, which resulted in $2.8 billion in property damage, 1150 injuries, and 161 deaths. Hurricanes and storms caused the most property damage of any MDE, with a median of $1 130 000 000 (IQR, $500 000 000-$3 880 000 000), whereas extreme heat MDEs rarely caused property damage.

### Rural vs Urban MDE Distributions and Health Care Capacity

Most weather events occurred in urban areas (6559 of 10 495 [62.5%]), and a large proportion of MDEs (151 of 201 [75.1%]) occurred in urban communities (Table 2). Urban MDEs accounted for 1616 deaths (median, 4 [IQR, 0-14] per event) and 15 499 injuries (median, 61 [IQR, 0-121]). Rural MDEs accounted for a total of 470 deaths (median, 9 [IQR, 2-16] per event) and 3501 injuries (median, 60 [IQR, 25-95] per event). Rural MDEs occurred most often in either ASPR region 4 (23 events [46%]) or region 6 (14 events [28%]).

**Table 2.** Demographics of MDEs vs Non-MDEs Between 2006 and 2021

Demographic	No. of events (%)^a^		
	Total (n = 11 159)	Non-MDE (n = 10 950)	MDE (n = 209)
Rurality			
Urban	6559 (62.5)	6408 (62.2)	151 (75.1)
Rural	3936 (37.5)	3886 (37.8)	50 (24.9)
Missing	664	656	8
ASPR region			
1	285 (2.6)	284 (2.6)	1 (0.5)
2	687 (6.2)	674 (6.2)	13 (6.3)
3	701 (6.3)	692 (6.4)	9 (4.3)
4	3076 (27.8)	3025 (27.8)	51 (24.5)
5	1376 (12.4)	1363 (12.5)	13 (6.3)
6	1792 (16.2)	1754 (16.1)	38 (18.3)
7	815 (7.4)	784 (7.2)	31 (14.9)
8	637 (5.7)	635 (5.8)	2 (1.0)
9	1357 (12.2)	1312 (12.1)	45 (21.6)
10	353 (3.2)	348 (3.2)	5 (2.4)
Missing	80	79	1

Abbreviations: ASPR, Administration for Strategic Preparedness and Response; MDE, major disaster event.

^a^ Percentages exclude missing data.

After adjusting for urban and rural populations, ASPR region urban counties had a total of 2.07 hospital beds per 1000 people, whereas rural counties had 1.50 beds per 1000 people (Table 3). The greatest difference between the proportions of urban vs rural populations and hospital beds was in ASPR region 5 (rural population, 19%, rural hospital beds, 12%) and region 7 (rural population, 31%, rural hospital beds, 23%). Additional information on populations, hospital beds, and land area densities are provided in the eTable in Supplement 1.

**Table 3.** Densities and Proportions of Rural and Urban Populations, Hospital Beds, and MDEs by ASPR Region

ASPR region	Population density^a^		Population-adjusted hospital bed density^b^		Proportion of regional population, %		Proportion of regional hospital beds, %		Proportion of regional MDEs, No. (%)	
	Urban	Rural	Urban	Rural	Urban counties	Rural counties	Urban counties	Rural counties	Urban counties	Rural counties
1	235.52	16.34	2.18	1.56	87	13	91	9	1 (100)	0
2	358.42	21.71	2.25	1.41	95	5	97	3	13 (100)	0
3	172.41	22.20	2.17	1.34	89	11	93	7	7 (88)	1 (12)
4	130.15	21.67	2.30	1.71	83	17	87	13	28 (55)	23 (45)
5	148.81	18.04	2.13	1.26	81	19	88	12	10 (77)	3 (23)
6	92.21	6.69	2.03	1.62	84	16	87	13	24 (63)	14 (37)
7	73.88	7.29	2.56	1.73	69	31	77	23	25 (81)	6 (19)
8	41.47	2.35	1.90	1.72	76	24	78	22	2 (100)	0
9	99.45	3.49	1.66	1.11	97	3	98	2	37 (95)	2 (5)
10	42.26	1.30	1.56	0.99	84	16	89	11	4 (80)	1 (20)

Abbreviations: ASPR, Administration for Strategic Preparedness and Response; MDE, major disaster event.

^a^ Population density calculated as No. of people per square kilometer.

^b^ Adjusted hospital-bed density is the proportion of hospital beds in a given urban or rural region divided by the population of that urban or rural region in 1000s of people.

### Regional MDE Distribution

**Figure 1.**
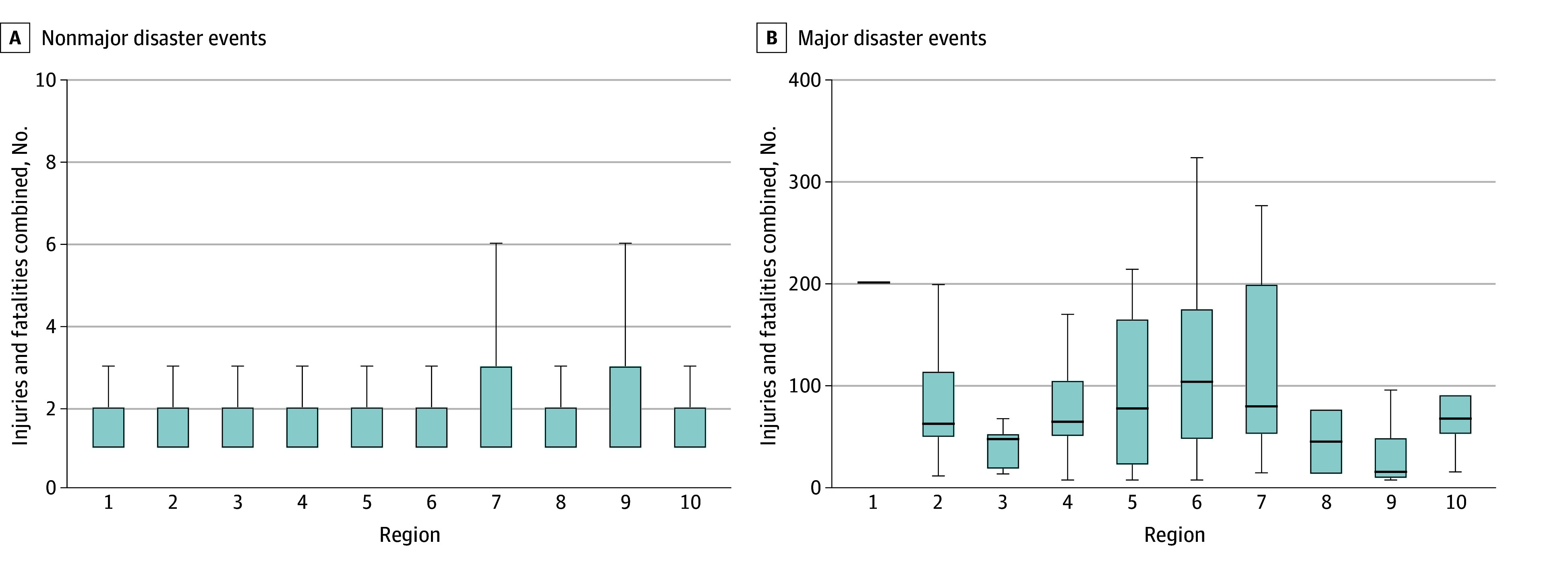
Combined Injuries and Fatalities From Nonmajor and Major Disasters by Administration for Strategic Preparedness and Response Region The lower and upper ends of the boxes are the first and third quartiles. The whiskers indicate 1.5 times the IQR added and subtracted, as appropriate, to the first and third quartiles. Outlier values are excluded. In panel B, the horizontal bars inside the boxes indicates the median.

**Figure 2.**
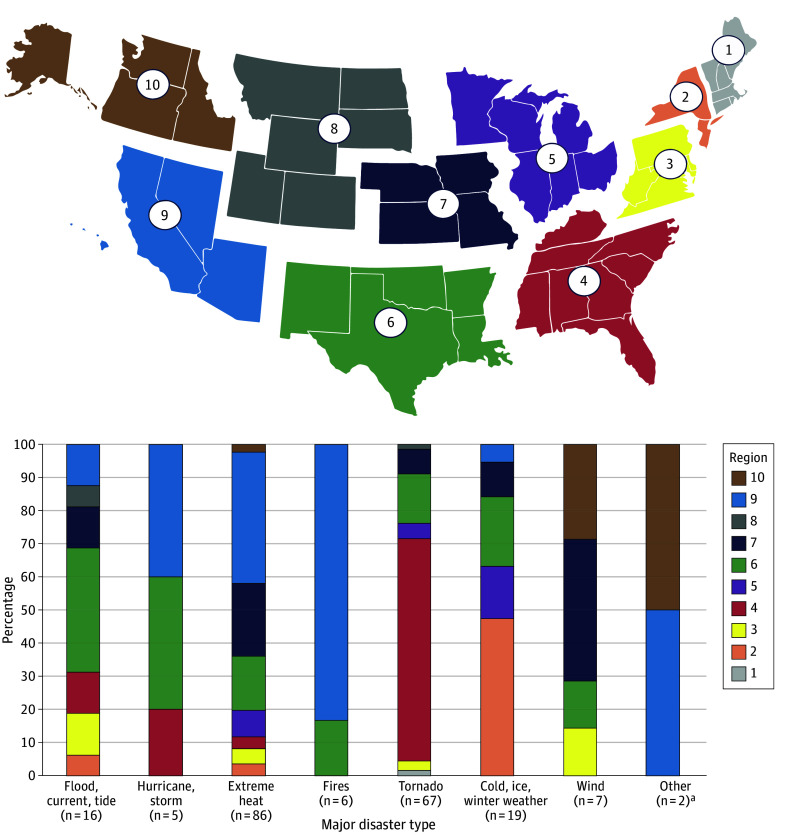
Proportion of Major Disaster Event Types Occurring in Each Administration for Strategic Preparedness and Response Region ^a^The other major disaster event in both regions was of debris flow (a type of severe landslide).

The MDEs had differing regional distributions, with 51 of 209 (24.5%) MDEs occurring in region 4 and 45 of 209 (21.6%) occurring in region 9 (Table 2). Comparing the number of MDEs that occurred in a region with the total number of weather events, regions 1 and 8 had the lowest proportion of MDE to weather events (1 of 285 [0.4%] and 2 of 637 [0.3%], respectively). Conversely, MDEs were 2.1% (38 of 1792) of weather events in region 6, 3.8% (31 of 815) in region 7, and 3.3% (45 of 1357) in region 9. Regions 5, 6, and 7 had the highest median number of injuries and fatalities from MDEs (Figure 1). In examining the regional distribution of MDE type, fires, wind, and hurricanes or storms were geographically concentrated, while extreme heat and floods affected regions equally across the US (Figure 2). Tornadoes had a general geographic predilection (region 4) but also occurred in 7 of 10 regions across the US.

## Discussion

The findings of this ecologic cross-sectional study show that MDEs impact regions across the US but vary in type and nature according to region. Although MDEs accounted for only 1.9% of all storm events in our analysis, they accounted for 46.1% of all storm-associated injuries and 22.4% of all storm-associated fatalities. Extreme heat and tornadoes were found to compose most of these events (73.2%), with the remainder of MDEs comprising a broad range of weather event types. The frequency of MDEs varied across the US, though a substantial proportion (46.1%) occurred in ASPR regions 4 (the Southeast) and 9 (the West). Together, the seasonal nature and geographic distribution of MDEs described in this study suggest that highly impacted regions may experience 2 or more MDEs in a single month. Despite the high MDE frequency and observed injuries and fatalities for both rural and urban events, rural areas showed a disproportionately lower hospital bed capacity than urban areas, even after adjusting for population differences. Given that 1 event could substantially strain state and regional resources, these data raise concerns about whether US health and trauma systems, specifically those in rural areas, are adequately prepared to care for patients with traumatic injuries after a major disaster.

Despite the morbidity and mortality caused by climate events, previous NOAA data have primarily characterized events by property damage and not by health outcomes.^10^ Our study found that the number of associated injuries and fatalities did not correlate with the property damage caused by a climate event. Apart from hurricanes and storms, no other category of MDEs in our study had a median or upper quartile of more than $1 billion in damage. In addition, our study found that some events had little to no property damage but were associated with substantial health morbidity through injuries and fatalities. This historic overreliance on classifying climate events and trends by property damage has created a system that risks misaligning regional health preparedness with potential threats.

Findings from previous clinical studies have suggested that climate-related injuries differ from standard traumatic injuries, with patients injured by wildfires experiencing higher mortality rates, longer stays in the hospital, and increased infections and those injured in tornado events requiring increased intensive care resources and surgical interventions.^11,12,13,14^ These studies also raise concerns that vulnerable populations who require specific medical resources, such as people living in mobile homes, older adults, and children, are at particular risk of severe injuries during climate events.^12,13,14^ As a result, following an MDE, health care systems need to be prepared not only for increased volumes of patients but also for patients with complex treatment requirements that may burden an already overextended health system. This is primarily a concern in rural and underserved areas that have disproportionally fewer hospital beds than urban areas, which may translate into a reduced surge capacity during MDEs.^15,16^ Policymakers should be aware of the influence of regionalized trauma care on rural populations during disaster events. To address these gaps, trauma and disaster planning must be aligned to ensure that surge capacity can be optimized within the state trauma system during an MDE and that necessary resources can reach affected areas.

A major barrier to climate-injury characterization is the lack of current linkages between climate and health data. The climate event data presented in this study are derived from the NOAA National Centers for Environmental Information, whereas national health statistics are primarily collected and maintained by the Centers for Disease Control and Prevention (CDC). The CDC fatality data are aggregated by month and year, making it difficult to correlate with specific climate events. Furthermore, CDC classifications of emergency department visits for injuries, hospitalizations, and causes of death are also based on *International Classification of Diseases* coding, which until 2019 did not include natural disaster injury codes.^17^ The classification of climate-related external causes by *International Classification of Diseases* code requires that the clinician responsible for the injured patient correctly lists the climate-associated code during their assessment. Thus, even for 2019 to 2023, when coding of climate events has been possible, the existing CDC health data likely underestimate the burden of climate-associated injuries and fatalities. The CDC provides some tools for disaster response, such as the Community Assessment for Public Health Emergency Response Toolkit.^18^ This tool is designed to be conducted at the household or community level and includes 1 question on injury. Other optional CDC disaster-related morbidity and mortality surveillance tools exist, but it is unclear in what scenarios health systems might deploy the various tools and what data have been collected.^19^ Future departmental collaboration and epidemiologic research on associations between weather events and injuries and fatalities are needed.

Our study also found that MDEs often occurred in rural areas. Currently, 14% of the US population (46 million individuals) live in rural communities.^20,21^ However, 35% of MDEs occur in rural areas, accounting for 23% of all MDE-associated fatalities and 18% of all MDE-associated injuries, despite the lower population density in rural areas compared with urban areas. Notably, the median number of fatalities per event in rural areas was more than double the median number of fatalities in urban areas (9 vs 4 per event), suggesting that rural communities may be more vulnerable to climate disasters. Between 2010 and 2021, 136 rural hospitals closed, which may explain some challenges with rural bed capacity and access to basic trauma care, accounting for a greater number of fatalities.^22^ Rural health care systems have historically lacked hazard mitigation plans, access to well-coordinated trauma care, planning for children’s interests, and the capability to rapidly transport injured patients to tertiary care centers.^16,23^ Rural housing and infrastructure are often not climate ready, which may result in greater vulnerabilities to certain climate events, such as extreme heat, flooding, and tornadoes.^24^ Power grid outages, which often co-occur with climate disasters, may exacerbate the negative health impacts of these events, especially in rural areas where power restoration can take days to weeks.^25^ As rural communities account for more than 70% of the landmass of the US, it is expected that severe storms will more often occur in these areas, making it critical to ensure preparedness in the rural communities.

Although monitoring the epidemiologic impacts of climate events that cause substantial injuries and fatalities is a necessary component of preparedness, there is currently no consensus definition in the US for what level of injury or fatality would merit reporting. Our definition of a major disaster, an event that causes at least 50 injuries or at least 10 fatalities, was adapted from a Delphi survey in South Korea.^3^ Given the differences between the Korean and US health systems, an important consideration is whether this definition appropriately applies to the US population. Future work to conduct a similar Delphi survey among climate, health, and trauma experts in the US is one opportunity to ensure the appropriate tracking of epidemiologic health outcomes following climate disasters. Such an effort might allow NOAA to report the frequency of MDEs annually or semiannually, as is done for property damage, and to better track and assess the health outcomes associated with climate-related events.

### Limitations

This study has some limitations. The ecologic study design, which focuses on population-level data at the ASPR region level, limits the examination of patient demographics, injury type, and clinical outcomes of any injury or fatality associated with climate events. As the SED is a weather event database, injury and fatality data were not subjected to the same validation used by health-based organizations, such as the CDC’s National Vital Statistics System. The SED data are derived from the National Weather Service, which receives inputs from various sources but is subject to underreporting. Reliance on different data sources may introduce a selection bias and misreporting of associated injuries and fatalities. Furthermore, reliance on municipalities to report fatalities associated with an event may result in undercounting. Prior research has suggested that the SED may underestimate deaths compared with FEMA and Red Cross reports but may have higher counts than National Vital Statistics data.^26^ However, FEMA and Red Cross data are limited to declared disasters, which limits the ability for a broader national assessment of these events. Overall, the challenges with these datasets underscore a need for increased linkage between health datasets, especially trauma registries and climate data. Improvements in documentation are needed for more accurate assessments of the association of traumatic injuries with climate events.

## Conclusions

This ecologic cross-sectional study found that MDEs have an outsized impact on all weather-related injuries and fatalities, accounting for almost half of all injuries and nearly 1 in 4 fatalities, despite their rare occurrence. These data should be integrated into hazard vulnerability analyses at the hospital, county, state, and regional level to improve national preparedness and reduce injuries associated with severe storms. Public tracking of these storms and their associated injuries and fatalities might allow for better characterization of the continued vulnerability of the US population due to climate change and ensure that the national health system has the capacity to respond to these events.

## Notes

Supplement 1. **eTable.** Rural and Urban Population, Hospital Bed Density, and MDE Proportion, by ASPR Region

Supplement 2.
Data Sharing Statement
