# Supplementary material for: Extreme Weather Injuries and Fatalities, 2006 to 2021
Source: JAMA Netw Open. 2024 Aug 26;7(8):e2429826. doi: 10.1001/jamanetworkopen.2024.29826 (PMC12634132; doi:10.1001/jamanetworkopen.2024.29826)
Supplement: Supplement 1. — eTable. Rural and Urban Population, Hospital Bed Density, and MDE Proportion, by ASPR Region [file jamanetwopen-e2429826-s001.pdf]

# Supplemental Online Content

Stephens CQ, Newton C, Kappy B, Melhado CG, Fallat ME. Extreme weather injuries and fatalities, 2006 to 2021. *JAMA Netw Open*. 2024;7(8):e2429826. doi:10.1001/jamanetworkopen.2024.29826

**eTable.** Rural and Urban Population, Hospital Bed Density, and MDE Proportion, by ASPR Region

This supplemental material has been provided by the authors to give readers additional information about their work.

© 2024 Stephens CQ et al. *JAMA Network Open*.

**eTable.** Rural and Urban Population, Hospital Bed Density, and MDE Proportion, by ASPR Region

| ASPR Region | Urban Population (in millions) | Rural Population (in millions) | Urban Land Area (in thousands of $km^2$) | Rural Land Area (in thousands of $km^2$) | Urban Hospital Beds (in thousands) | Rural Hospital Beds (in thousands) | Urban Hospital Bed Density (beds per thousand people) | Rural Hospital Bed Density (beds per thousand people) |
|---|---|---|---|---|---|---|---|---|
| Region 1 | 12.21 | 1.76 | 51.82 | 107.87 | 26.63 | 2.74 | 26.63 | 2.74 |
| Region 2 | 27.98 | 1.37 | 78.06 | 63.03 | 62.96 | 1.93 | 62.96 | 1.93 |
| Region 3 | 27.86 | 3.31 | 161.57 | 149.20 | 60.38 | 4.45 | 60.38 | 4.45 |
| Region 4 | 55.26 | 11.47 | 424.54 | 529.49 | 127.18 | 19.58 | 127.18 | 19.58 |
| Region 5 | 43.10 | 9.85 | 289.63 | 545.91 | 91.85 | 12.38 | 91.85 | 12.38 |
| Region 6 | 35.71 | 6.87 | 387.29 | 1027.83 | 72.58 | 11.11 | 72.58 | 11.11 |
| Region 7 | 9.83 | 4.38 | 133.02 | 600.41 | 25.17 | 7.55 | 25.17 | 7.55 |
| Region 8 | 9.30 | 2.97 | 224.15 | 1261.10 | 17.69 | 5.12 | 17.69 | 5.12 |
| Region 9 | 49.29 | 1.76 | 495.64 | 503.56 | 81.69 | 1.94 | 81.69 | 1.94 |
| Region 10 | 12.10 | 2.27 | 286.20 | 1738.91 | 18.92 | 2.24 | 18.92 | 2.24 |

© 2024 Stephens CQ et al. *JAMA Network Open*.
