# Supplementary material for: Extreme Weather Injuries and Fatalities, 2006 to 2021
Source: JAMA Netw Open. 2024 Aug 26;7(8):e2429826. doi: 10.1001/jamanetworkopen.2024.29826 (PMC12634132; doi:10.1001/jamanetworkopen.2024.29826)
Supplement: Supplement 2. — Data Sharing Statement [file jamanetwopen-e2429826-s002.pdf]

Data Sharing Statement

Stephens. Extreme Weather Injuries and Fatalities, 2006 to 2021. *JAMA Netw Open*.
Published August 26, 2024. doi:10.1001/jamanetworkopen.2024.29826

Data
Data available: No
